# Complete root specimen of plants grown in soil-filled root box: sampling, measuring, and staining method

**DOI:** 10.1186/s13007-021-00798-3

**Published:** 2021-09-20

**Authors:** Takuya Koyama, Shun Murakami, Toshihiko Karasawa, Masato Ejiri, Katsuhiro Shiono

**Affiliations:** 1 School of Agriculture, Utsunomiya University, 350 Mine-machi, Utsunomiya, Tochigi 321-8505 Japan; 2 Central Region Agricultural Research Center (Kanto, Tokai and Hokuriku Regions), National Agriculture and Food Research Organization (NARO), 2-1-18 Kannondai, Tsukuba, 305-8666 Japan; 3 Graduate School of Bioscience and Biotechnology, Fukui Prefectural University, 4-1-1 Matsuoka-Kenjojima, Eiheiji, Fukui 910-1195 Japan

## Abstract

**Background:**

Detailed datasets containing root system and its architecture in soil are required to improve understanding of resource capture by roots. However, most of the root study methods have paid little attention to make and preserve whole root specimens. This study introduces root system sampling equipment that makes the entire root specimen with minimum impairment and without displacement of the spatial arrangement of the root system in root boxes. The objectives are to assess: whether the equipment can rapidly sample the entire root system; whether root surface area is measurable from a scanned digital image of the root specimen; and whether staining of the entire root specimens would provide multidimensional visual information on the interaction between soil and physiological function of root system architecture (RSA). For validation, we examined the root response of two soybean cultivars to arbuscular mycorrhizal (AM) inoculation and the effect of waterlogging stress on the physiological activity of buckwheat RSA.

**Results:**

The root boxes allowed soybean and buckwheat plants to grow uniformly across the replications. Both species showed significant differences between cultivars and/or among treatments in shoot and root traits. The equipment enabled to sample the whole-root specimens of soybean and buckwheat, where the tips of the fine roots were alive (diameter < 0.2 mm). Also, the whole root specimens of soybean were made in about 7 min. The root surface area calculated from the scanned soybean specimens showed a significant correlation with that calculated from the roots spread out in water (a common method). Staining of the soybean root specimens enabled us to observe the localized root proliferation induced by AM colonization. Moreover, staining of the buckwheat root specimens made it possible to examine the respiratory activity of each root at different depths.

**Conclusions:**

The present method realized: fast and accurate production of the whole root specimen and precise calculation of the specimens’ root surface area. Moreover, staining of the root specimens enabled analyzing the interaction between soil and physiological function of RSA. The evaluation of root traits, using our methods, will contribute to developing agronomic management and breeding program for sustainable food production.

**Supplementary Information:**

The online version contains supplementary material available at 10.1186/s13007-021-00798-3.

## Background

Developing methods that generate detailed datasets containing the root system and its architecture in the specific soil environment enhance crop cultivation techniques that contribute to sustainable food production [1, 2] and promote crop breeding adapted to stressful soil environments [3–5]. Root system consists of component roots that are different in emergence [6, 7], morphology [8–11], anatomy [12], and function [13], called “heterorhizy” [11, 14, 15]. The heterorhizy includes a taproot, adventitious roots, and S- and L-type lateral roots (LRs) for dicot crops, and seminal root(s), nodal roots, and their S- and L-type LRs for monocot crops [8–10]. These component roots show different functions with aging [16]. They interact with one another under certain soil environments and influence plant functions that contribute to performance and eventually yield [17].

On the other hand, the soil is also heterogeneous. Soil comprises a complex distribution of particles of different sizes, compositions, physical properties, airspaces, variations in nutrient and water availability, and microbial diversity. Such physical, chemical, and biological properties of soil can vary depending on spatial and temporal scales. Under these heterogeneous conditions, plants can adjust the placement of their new roots to capture the resources in response to the soil environment, which is known as “root plasticity” [11, 18, 19]. Thus, a better understanding of resources captured by plant roots requires detailed datasets that include the root system architecture (RSA) and the heterogeneous soil conditions.

Root box, also called rhizobox, grows plants in transparent polyvinyl chloride (PVC), wood, glass plate, or metal boxes and has been widely used for soil-based root researches [20–22]. Recently, some platforms using root boxes such as GLO-Roots and GrowScreen-Rhizo have been established for high-throughput root phenotyping [23, 24]. There are two ways to describe the root system in root boxes. One is to record the visible roots on the outer surface of the root box by scanning or taking pictures of the root box surface or manually drawing the visible roots on transparent sheets [23–26]. The other is to drive a needle board into the substrate in the box, remove the substrate from the needle board, and measure the root phenotypic traits on the needle board [21, 22].

However, most of the methods of root studies have paid little attention to producing a complete root specimen. In 1987, Kono et al. [27] developed a revised pinboard method that made a whole root specimen with minimum structural impairment and disturbance to study the interaction between the morphology, topology, and placement of each component root and soil environment in detail. The method requires a root box with removable side panels (length (L) 25 cm × width (W) 2 cm × height (H) 40 cm), a pinboard with 960 nails in a grid pattern at 10 mm intervals, and a double-folded polyethylene sheet with 960 holes of 5 mm diameter on both sides aligned with the nail positions on the pinboard. With this equipment, the whole root specimen is collected according to the following procedure. (1) After cultivating plants, remove the side panel of the root box in which the plant was grown with soil, push a pinboard covered with one side of the sheet into the soil in the box, flip the pinboard, and remove the root box from the soil containing roots. (2) Wash away the soil gently by a hose with a showerhead and cover the other side of the sheet to trap the root system in the sheet. (3) Pull out the root system from the pinboard with the sheet and preserve the root system between sheets. Using the method, researchers examined the different response of the component roots to the soil environment, including soil moisture [8, 28–38], soil compaction [39–41], microorganism [42–48], and allelopathic substances [49]. These studies have led to the detection of the quantitative trait loci (QTLs) associated with LR plasticity and root aerenchyma development for fluctuating soil water stress [32, 33, 36] and the development of agricultural practices such as “crack fertilization” on soybean root nodulation: a combination of deep layer inoculation with rhizobia on granular volcanic soil or biochar and subsoiling during plant growth [43, 44, 50]. In addition, the findings were consistently confirmed under field conditions [29, 30, 34, 38, 43, 44].

However, the use of the revised pinboard method has the following possible disadvantages. Firstly, it is time-consuming to make the sheet with 960 holes of 5 mm diameter and to align the pinboard with the root boxes. Among the apparatuses required for the revised pinboard method, the sheet with 960 holes of 5 mm diameter is the most important to sample and preserve the whole root specimen. Since the sheets are made manually using a hole-puncher, it takes a lot of time and labor. It is necessary to make the same number of sheets, when processing many plants to examine the interaction between the RSA and soil environment. Also, the whole root specimen sampling requires about fifteen minutes per sample per person [27]. This may be because it takes careful work to push the nail board onto the root boxes. Secondly, to capture the optimum contrast of roots, especially LRs, from the root specimens sandwiched between the sheets, it was necessary to dye the roots before imaging [27, 51].

To overcome these disadvantages, we developed root system sampling equipment. It does not require the time-consuming procedure of making the sheets with 960 holes and facilitates the fast and accurate alignment of the nail board to the root boxes. In addition, using the equipment, the diameter of holes on the sheets became smaller than 5 mm: around 0.014 cm^2^ as projected area. It is expected to provide an analysis of root surface area accurately from the root specimen. Therefore, the objectives of this study are to assess whether the whole root system can be sampled rapidly by the equipment with minimum impairment and disturbance to its structure and whether the entire root surface area can be measured from the scanned digital image of the root specimen sampled with the equipment. For validation, soybean (*Glycine max* (L.) Merr.) and common buckwheat (*Fagopyrum esculentum* Moench) plant species were sampled with the equipment, and the scanned digital image of the soybean root specimen was analyzed with the commercially available image analysis system.

Further, staining of the whole root specimen can provide multidimensional visual information on the interaction between soil and the physiological function of RSA. The objectives include evaluating the possibility. The study examines the root response of two soybean cultivars to arbuscular mycorrhizal (AM) inoculation and the effect of waterlogging stress on the physiological activity of the root system of buckwheat plants.

## Methods

### Preparation of root box

**Fig. 1:**
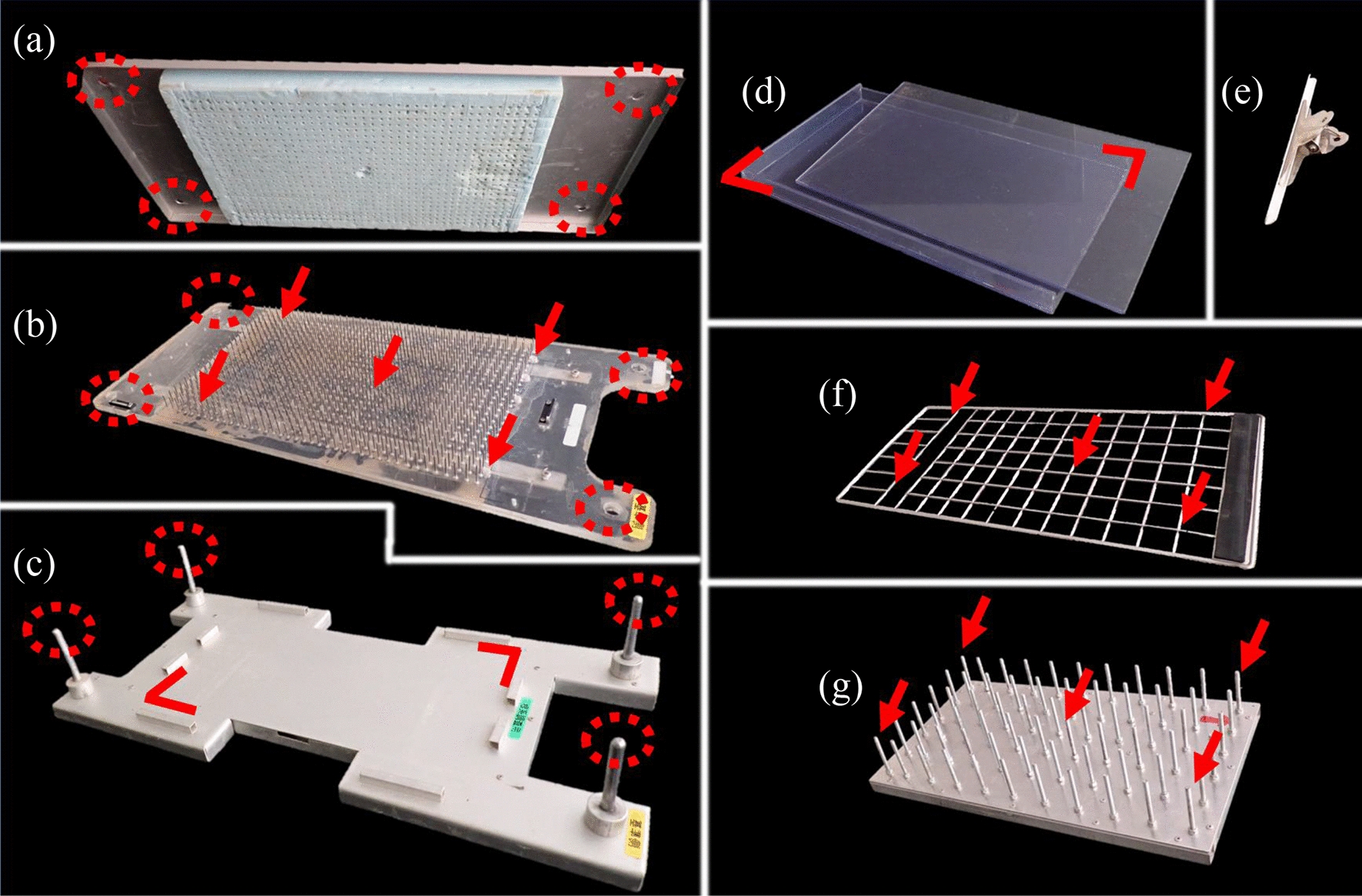
Root system sampling equipment (**a**–**c**, **e**–**g**) and root box (**d**). Root system sampling equipment is composed of a urethane foam board (**a**), a nail board (**b**), a workbench with aluminum square pipes and guide bars (**c**), a clip (**e**), a stainless steel grid (**f**), and a pressing plate (**g**). Dotted circles in **a** and **b** indicate holes for the guide bars (dotted circles in **c**). The root box is placed on the workbench along the aluminum square pipes (brackets in **c**). Arrows in **b** indicate the holes for protrusions of the grid pressing plate (arrows in **g**). The protrusions on the pressing plate (arrows in **g**) push the intersections of the grid (arrows in **f**) to separate the whole root specimen sandwiched between sheets from the equipment

**Fig. 2:**
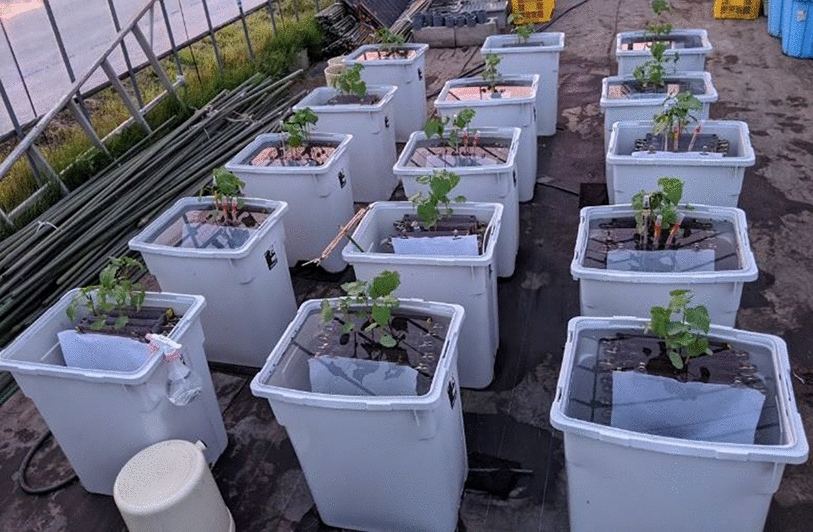
Buckwheat plants cultivated under either control, 3-day, or 6-day long waterlogged conditions. The containers (L 365 mm × W 485 mm × H 600 mm) containing root boxes were placed in a greenhouse to protect the plants from rain under natural light conditions. Each container can contain up to six root boxes. In the waterlogging treatments, the soil was waterlogged to approximately 30 mm above the soil surface

Root boxes were prepared according to Kono et al. [27] and Kano-Nakata et al. [51]. The boxes were made of transparent PVC of 5 mm thickness (Fig. 1d) with the dimensions L 25 cm × W 2 cm × H 40 cm. Only a wall of one side of the box (L 25 cm × H 40 cm) was removable. The wall was mounted to the main box and secured with six clips per box. The root boxes were filled with soils as follows: after filling the root box evenly with soil, the root box was dropped from a height of 10 cm to the ground surface three times, and then the soil was added to the root box again and dropped once from a height of 10 cm to the ground surface. This soil packing procedure is recommended to keep the soil bulk density constant [27]. A day before sowing, the soil in the boxes was completely submerged in tap water for around 30 s. Then, the boxes were erected vertically overnight to allow water to drain to the field capacity through the gap between the boxes and the removable walls. Before sowing, about 4 cm of soil layer was cultivated with tweezers. Then, the three seeds were sown with the radicle facing downwards. The seeds of soybean were sown at 3 cm from the soil surface and 5 cm apart from each other. Those of buckwheat were sown at 1.5 cm from the soil surface and 2 cm apart from each other. Seedlings were thinned into one plant in each box after the plant was established. Thinning was done by cutting the stems. Similar plants in terms of size between the replications were chosen. The boxes were placed in a greenhouse to protect the plants from the rain under natural light conditions (Fig. 2). These boxes were covered with aluminum foils in 2017. However, since aluminum foil damages PVC, they were covered white felt with 1 mm thickness in 2020. These covers were necessary to prevent light penetration to the roots and to prevent algal growth on the root box.

### Root system sampling

Root system sampling equipment is comprised of a nail board with stainless nails (2.7 cm length), a workbench with aluminum square pipes and guide bars, a urethane foam board, a clip, a stainless steel grid, and a grid pressing plate (Fig. 1). The following procedure is shown in Additional file 1: Figure S1 and Additional file 2: Movie S1.

During root sampling, we measured each time spent for “preparation of root washing”, “root washing”, and “removal of the sheet from the nail board”. Preparation of root washing included the following procedures. A folio of polyethylene sheet (L 320 mm, H 530 mm × 2) was made by cutting out both sides of commercially available polyethylene bags (L 380 mm, H 530 mm, W 0.04 mm). The thickness of polyethylene bags was 0.04 mm: if it is thicker, it is difficult for the nail to penetrate; and if it is thinner, it is difficult to handle the root specimen as it is soft and limp. The nail board, with the nails facing up (Fig. 1b), and the grid (Fig. 1f) were installed on the workbench (Fig. 1c) in this order along the guide bars at the four corners of the workbench (Additional file 1: Figure S1.1). The clip (Fig. 1e) secured the folds of the polyethylene sheet to the nail board. One side of the folded polyethylene sheet was opened, while the corners of the remaining side were pressed into the nails with fingers. Then, along the guide bars, the remaining side of the sheet was pushed into the nail board with the urethane foam board (Fig. 1a). The nail board with one side of the sheet and the grid were removed from the workbench. The root box with the removable wall on the top (Fig. 1d) was submerged in water during the above procedures. The clips were removed, and the removable wall was slid upwards along the box (Additional file 1: Figure S1.2). The box, then, was placed on the workbench along the aluminum square pipes (Additional file 1: Figure S1.3). The nail board with one side of the sheet and the grid was pressed against the soil in the box along the guide bars of the workbench. After pressing, we turned the equipment above besides the workbench upside down, and the root box was removed downward with the soil, leaving the soil profile on the nail board (Additional file 1: Figure S1.4).

Root washing included the following procedure. The soil on the nail board was washed away gently by a hose with a showerhead (Additional file 1: Figure S1.5) from the bottom of the box. If water were splashed from the top of the box, the roots would have dropped with the soil. The residues were removed with tweezers.

Removal of the sheet from the nail board included the following procedure. The nail board with the root system was placed along the guide bars of the workbench (Additional file 1: Figure S1.6). The four corners of the remaining side of the sheet were pushed with fingers, and the sheet was pushed into the nail board with the urethane foam board. Thereby, the root system was sandwiched between the sheets. The nail board with folded sheets and the grid were removed from the workbench and turned upside down. The protrusions of the grid pressing plate (Fig. 1g) were inserted along the holes of the nail board, pressing the intersections of the grid. Then, the sheet along with the whole root system was removed from the nails with the grid (Additional file 1: Figure S1.7).

### Plant material and growth conditions

Two experiments were conducted to address the objectives. Experiment 1 intended to assess whether total root surface area could be measured from the scanned digital image of the soybean root specimen sampled by the equipment. Both experiments 1 and 2 intended to assess: whether the whole root system could be sampled rapidly by the sampling equipment with minimum impairment and disturbance to its structure and whether staining of the entire root specimen provides multidimensional visual information on the interaction between soil and physiological function of RSA.

Experiment 1 examined the root response of two soybean cultivars (*Glycine max* (L.) Merr.) to AM inoculation. The two cultivars were Nattoukotsubu and Tachinagaha, the popular cultivars in the Kanto region, Japan. Ten grams of either an AM fungal inoculum R-10 (Idemitsu Kosan Co., Ltd., Tokyo), which had a positive impact on soybean yield under field conditions [52, 53], or the sterilized one with 0.14, 0.44, and 0.15 g kg^−1^ of powdered ammonium sulfate, calcium superphosphate, and potassium chloride, respectively, were thoroughly mixed into the sterilized volcanic ash soil (particle size: 1–3 mm, Shibanome-tsuchi, Shidara Co. Ltd., Tochigi, Japan). The inoculum consisted of spores, extraradical hyphae, and chopped colonized roots with a crystalline silica carrier. The soil was filled in the root box, as stated above. Then, the root boxes containing the soil were submerged in water containing bradyrhizobia (*Bradyrhizobium japonicum* USDA110 ca. 10^3^ cells mL^−1^). On August 25, 2017, a day after the submergence, the seeds were surface-sterilized and sowed. Watering was done by the submerging method mentioned above every week. Micronutrients were supplied with 50 mL of the solution to each root box on September 4, 19, and October 3, 2017. The micronutrients included: CaCl_2_·2H_2_O 262.0, MgSO_4_·7H_2_O 245, FeEDTA·3H_2_O 43.9, MnSO_4_·5H_2_O 1.43, ZnSO_4_·7H_2_O 0.25, CuSO_4_·5H_2_O 0.25, H_3_BO_3_ 0.25, Na_2_MoO_4_·2H_2_O 0.05, and CoCl_2_·6H_2_O 0.03 (mg L^−1^). The initial pH of the solution was about 6.25. Four replicate root boxes were prepared for each cultivar and treatment. The cultivars and treatments were arranged in a completely randomized design. The plants were sampled on October 16, 2017.

Experiment 2 examined the effect of waterlogging stress on the physiological activity of the buckwheat root system. Common Japanese buckwheat (*Fagopyrum esculentum* Moench cv. kitawasesoba) was used in this experiment because it is susceptible to waterlogging stress [54]. The root boxes were filled with potting soil containing volcanic ash soil, humus, and peat (particle size: 0.5 to 3 mm, 200 mg N L^−1^, 2500 mg P_2_O_5_ L^−1^, 200 mg K_2_O L^−1^, Kumiai Nippi engeibaido, Nihon Hiryo, Tokyo). The seeds were sowed on May 12, 2020. Sowing was conducted following the procedure mentioned above. When seedlings had the 3rd leaf (May 30, 2020), the boxes went through either 3 or 6-day waterlogging treatments (W3 or W6). In the waterlogging treatments, the plants were flooded to ~30 mm above the soil surface, whereas five root boxes remained as drained controls (C) and were watered with tap water by the submerging method mentioned above every 2 or 3 days. After the waterlogging treatment, the root boxes were drained and subsequently watered in the same way as stated above. Five replicate root boxes were prepared for each treatment. The treatments were arranged in the randomized complete block design. The plants were sampled on June 12, 2020.

The air temperature and relative humidity of each experiment were shown in Additional file 3: Figure S2. They were cited from the nearest observatory of each experimental site: central region agricultural research center, NARO in Tsukuba for experiment 1 and Utsunomiya university in Utsunomiya for experiment 2 (Aerological Observatory in Tsukuba, Ibaraki, Japan and Utsunomiya local meteorological office in Utsunomiya, Tochigi, Japan, Japan Meteorological Agency, www.data.jma.go.jp).

### Measurement

In experiment 1, each time spent for “preparation of root washing”, “root washing”, and “removal of the sheet from the nail board” was measured. Then, the root sample was stored, scanned, cleared, and stained to analyze the root response to AM inoculation. For preservation, one side of the sheet sandwiching the root was peeled with care. Then, the sampled root systems were sprayed with 70% ethanol and sandwiched again between the sheets. They were preserved in plastic bags with zippers until the following analyses. The sampled AM root systems were cut into nine rectangular sheets (L 13 cm × W 8 cm) to compare the root surface area directly calculated from the root specimen of soybean plants and the surface area calculated from the roots spread out on a Plexiglass tray filled with enough tap water to completely cover the roots.

For scanning, the A3 (L 297 mm × W 420 mm) scanner (ES 10000G, Seiko Epson Corp., Nagano, Japan) equipped with the scanner transparency unit (ESA3FLU3, Seiko Epson Corp., Nagano, Japan) was used. Firstly, tap water was sprayed on the scanner glass, the sheet sandwiching the roots was placed on top, one side of the sheet was carefully peeled off without moving the roots, and air bubbles between the remaining sheet and the scanner glass were removed with fingers. Then, the root system was scanned at a resolution of 600 dpi. After scanning the roots, the remaining sheet was removed, and then the roots were spread out on a Plexiglas tray filled with enough tap water to cover the surface of the roots and scanned again. The images were saved in TIFF format and analyzed using an image analysis system (WinRHIZO Pro 2017, Regent Instruments, Inc., Quebec City, QC, Canada). The following settings of WinRHIZO Pro were used for the analysis: the automatic threshold was selected to distinguish roots from the background, and the objects smaller than 0.02 cm^2^ were treated as debris and removed from the analysis with the system of “debris and rough edges filters”.

In order to analyze the AM colonization of the roots, the roots sandwiched between each rectangular sheet were cut into every 5 mm in length and cleared with 10% KOH and stained with trypan blue in lactoglycerol [55]. Around 20 stained root samples were randomly collected from each rectangular sheet, and the percentage of root colonization by AM hyphae was estimated by the magnified intersections method [56]. In each rectangular sheet, ~34 intersections were examined under a compound microscope at 100 × magnification. Thus, more than 250 intersections were examined for each plant. The shoots were dried at 80 °C for 72 h, weighed, ground with a mill, and digested with a mixture of nitric acid/perchloric acid. Phosphorus concentration in the digests was determined with the vanadomolybdate-yellow assay [57].

In experiment 2, to determine the respiratory activity of buckwheat roots, 2,3,5-Triphenyl-2*H*-tetrazolium chloride (TTC) was used. TTC is a redox indicator used to analyze cellular respiration. It is a colorless compound that turns red when reduced by dehydrogenases in living cells. TTC (WAKO, Osaka, Japan) was dissolved in 0.1 M sodium phosphate buffer (pH 7.0) to a final concentration of 0.6% (w/v). Soon after sampling the whole root specimen, it was placed on the flat surface with one side of the sheet peeled off, covered with a paper towel (Kimtowel, NIPPON PAPER CRECIA CO., LTD., Tokyo, Japan), and sprayed with TTC solution. They were incubated at 40 °C for 45 min and scanned with the A3 scanner (24-bit Colour mode, 600 dpi; DS-G20000, Seiko Epson Corp., Nagano, Japan). Root tips that turned red were considered alive and counted. After scanning in color mode, the roots were scanned again with a scanner equipped with a transparency function at the 600 dpi resolution. The following settings of WinRHIZO Pro were used for the analysis: a grey level threshold of 240 was selected to distinguish roots from the background, and the objects smaller than 0.02 cm^2^ were treated as debris and removed from the analysis, using the system of “debris and rough edges filters”.

The study measured xylem sap to estimate the physiological activity of the entire root system. A 1.5 ml microtube stuffed with cotton was weighed. Hypocotyls were cut with a sharp razor at the height of 5 cm above the soil surface. Just after cutting the hypocotyl, the microtube containing cotton was attached to the cut surface of the stem. After 1 h, the microtubes containing cotton were collected and weighed again. The xylem sap was calculated by the weight differences of the cotton before and after the procedure. The shoots were dried at 80 °C for 72 h and weighed.

### Statistical analysis

All statistical analyses were performed by statistical software (JMP 12.2, SAS Institute Inc., USA). The data were analyzed by analysis of variance (ANOVA) at significant levels of *p* < 0.001, 0.01, and 0.05 and the Tukey HSD test at a significance level of *p* < 0.05. Simple linear regression models were applied to analyze correlations among parameters at significant levels of *p* < 0.001, 0.01, and 0.05.

## Results

**Fig. 3:**
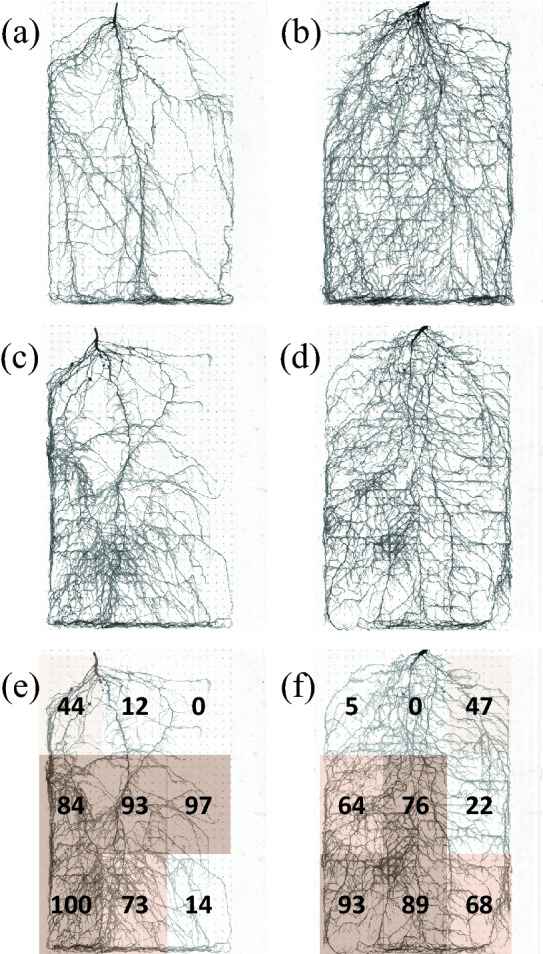
NM (**a**, **b**) and AM (**c**–**f**) whole root specimens of two soybean cultivars: Nattoukotsubu (**a**, **c**, **e**) and Tachinagaha (**b**, **d**, **f**). Each specimen was cut into 9 rectangular sheets, as shown in **e** and **f**. Root colonization by AM hyphae was measured from roots in each rectangular sheet. Numbers in **e** and **f** indicate the root colonization in each location. Shaded colors are darkened every 25%

**Fig. 4:**
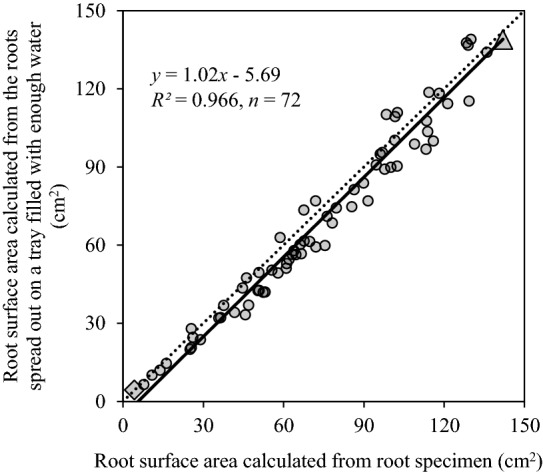
Correlation between the root surface area calculated from the root specimen of soybean plants and the roots spread out on a Plexiglas tray filled with enough tap water. The data were obtained from the rectangular sheets cut from the whole root specimens as shown in Fig. 3**e**, **f**. The dotted line indicates a one-to-one line. The images of filled triangle and filled diamond were analyzed with WinRHIZO and shown in Additional file 4: Figure S3

**Fig. 5:**
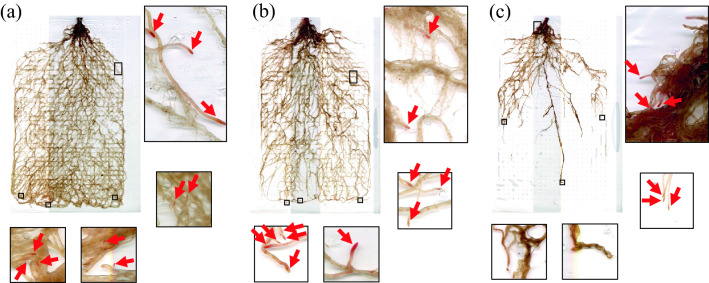
Effects of different duration of waterlogging stress on TTC stained whole root specimens of buckwheat plants: **a** control plant; **b** 3-day long waterlogged plant; and **c** 6-day long waterlogged plant. The frames on the whole root specimens indicate the location of close-up sections. Arrows indicate root tips that turned red. They were considered to be alive and counted in Table 2

In both experiments, the root sampling equipment allowed fast and accurate making the whole root specimen with minimum impairment and disturbance to its structure (Figs. 3, 5). Also, root surface area was calculated accurately from the root specimens of soybean plants (Fig. 4). The whole root systems of soybean plants were sampled within 7 min and 7 s ± 43 s (± *SE*) (*n* = 3). Preparation of root washing, root washing, and removal of the sheet from the nail board took about 2, 4, and 1 min, respectively. Besides, in experiment 1, there was a significantly positive correlation between the calculated root surface area from the root specimen of soybean plants and that calculated from the roots spread out on a Plexiglas tray filled with enough tap water (*p* < 0.001) (Fig. 4). However, due to the overlapping of roots (Additional file 4: Figure S3), the root length was underestimated and the average root diameter was overestimated (Additional file 5: Figure S4).

In experiment 1, after staining these whole root specimens, the study examined the root and shoot responses of soybean plants to AM inoculation. Table 1 summarizes the effect of AM fungal inoculation on the shoot and root traits of two soybean cultivars. There was a significant interaction between cultivars and inoculation treatments in shoot dry weight, root surface area, and phosphorus uptake. The shoot dry weight and root surface area of Nattoukotsubu tended to increase by inoculation, while those of Tachinagaha tended to decrease. Phosphorus uptake of AM inoculated Nattoukotsubu plants was five times higher than non-inoculated (NM) ones, whereas that of AM inoculated Tachinagaha plants was just 1.15 times higher than NM ones. The root colonization by AM hyphae of Nattoukotsubu was significantly higher than Tachinagaha. Also, the effect of AM colonization on the localized root proliferation was observed in Fig. 3. The root developed well where the AM colonization was high. There were significantly positive correlations between AM colonization and the deviation of root surface area in each of the nine rectangular sheets (Additional file 6: Figure S5). AM root-external hyphae were observed in some parts of the root specimen (Additional file 7: Figure S6), which was more frequent in Nattokotsubu than Tachinagaha (Data not shown).

**Table 1:** The effect of AM fungal inoculation on shoot dry weight, root surface area, phosphorus uptake, and root colonization by AM hyphae of two soybean cultivars of Nattoukotsubu and Tachinagaha

Cultivar	AM inoculation	Shoot dry weight	Root surface area	Phosphorus uptake	Root colonization by hyphae
		(g plant^−1^)	(cm^2^)	(g plant^−1^)	(%)
Nattoukotsubu	−	0.51 ± 0.06	439 ± 6	0.04 ± 0.00	
	+	0.92 ± 0.12	549 ± 37	0.20 ± 0.05	66.4 ± 12.9
Tachinagaha	−	2.07 ± 0.13	1013 ± 42	0.13 ± 0.01	
	+	1.58 ± 0.15	847 ± 60	0.15 ± 0.03	41.4 ± 14.2
Cultivar (C)		***	***	ns	*
Inoculation (I)		ns	ns	**	
C × I		**	**	*	

Values are means (*n* = 4). The percentage data of root colonization by AM hyphae were arcsine-transformed to normalize the distribution. ANOVA: ns, not significant; *p < 0.05; **p < 0.01; ***p < 0.001

In experiment 2, the study examined the effect of waterlogging stress on the respiratory activity of the buckwheat root system. The shoot dry weight and root surface area were significantly depressed as the waterlogging treatment duration became longer (Table 2). Although the buckwheat roots were small in diameter: the average diameter of buckwheat plants was around 0.3 mm and about 60% of total roots were classified as thinner than 0.2 mm in diameter (Additional file 8: Figure S7), the entire root system with live root tips was sampled and stored (Fig. 5). Thereby, it enabled the TTC staining of soil-grown roots. Most of the root tips of C were stained with TTC and turned red, but those of W6 were not stained. Additionally, the number of TTC stained root tips of W6 was significantly fewer than that of C and W3 (Table 2). The xylem sap of W6 was also significantly lower in weight than those of C and W3. There was a significantly positive correlation between the number of TTC stained root tips and xylem sap (Additional file 9: Figure S8).

**Table 2:** Effects of different duration of waterlogging treatment on shoot dry weight, root surface area, TTC stained root tip number, and xylem sap of buckwheat plants

Treatment	Shoot dry weight		Root surface area	TTC stained root tip	Xylem sap
	(g plant^−1^)		(cm^2^)	(no. plant^−1^)	(g plant^−1^ h^−1^)
C	4.84 ± 0.48^a^	1514 ± 108^a^		145 ± 15.9^a^	1.45 ± 0.16^a^
W3	2.75 ± 0.32^b^	923 ± 102^b^		120 ± 10.1^a^	1.46 ± 0.09^a^
W6	1.03 ± 0.19^c^	172 ± 36.9^c^		39.2 ± 7.11^b^	0.07 ± 0.04^b^

C, control; W3, 3-day long waterlogging treatments; W6, 6-day long waterlogging treatments. Values are means (*n* = 5). The different letters indicate significant differences among the treatments (Tukey HSD test, *p* < 0.05)

## Discussion

The present method displayed fast and accurate production of the whole root specimen with minimum injury or displacement of RSA, precise calculation of root surface area from the sample, and detailed analysis of root-soil interactions by staining the entire root specimen.

Firstly, the root sampling equipment allowed the fast and accurate making of the whole root specimen with minimum injury and displacement of RSA (Figs. 3, 5). Kono et al. [27] reported that the revised pinboard method required around 15 min to sample the whole root specimen. This study cut sampling time almost in half, 7 min, as shown in the sampling movie of the maize plant (Additional file 2: Movie S1). Among the sampling process, root washing time took up about half of the total sampling time, 4 min. The time required for root washing depends on soil types: it takes longer for the soil containing plant residues while shorter for sand. Kono et al. [27] used sandy loam soil sieved through 3 mm mesh. We used volcanic ash soil (particle size: 1.0–3.0 mm) and potting soil (particle size: 0.5–3.0 mm), indicating that the time required for root washing would be comparable. Among the sampling equipment, the aluminum square pipes on the workbench facilitated the fast and accurate alignment of root boxes, and the guide bars also assisted the alignment of the sheets, the nail board, and the urethane foam board. The guide bars ensured that the roots and the holes on the sheet were aligned at all times. It enabled the batch processing of WinRHIZO and the other root analysis software implemented with the batch analysis and reduced time for analyzing images.

The average diameter of buckwheat plants was around 0.3 mm and about 60% of total roots were classified as thinner than 0.2 mm in diameter (Additional file 8: Figure S7). Despite the thinness of the buckwheat roots, the whole root system with live root tips was sampled. It indicated that this method could be applied to other crops with thin component roots. For example, the average diameters of component roots of different crop species are as follows: in rice plants, the L-type LRs ranged between 0.05–0.2 mm, the S-type LRs ranged 0.05–0.13 mm; in maize plants, the L-type LRs ranged between 0.3–0.41 mm, the S-type LRs ranged between 0.15–0.4 mm [9, 39]; in soybean plants, the L-type LRs ranged between 0.28–0.73 mm, the S-type LRs ranged between 0.24–0.46 mm [8]; in Job’s tears plants, the S-type LRs ranged between 0.3–0.34 mm, the L-type LRs ranged between 0.14–0.29 mm; in sorghum plants, the L-type LRs ranged between 0.21–0.45 mm, the S-type LRs ranged between 0.1–0.27 mm [39]. These diameters differed depending on the growth duration and the soil environment, such as soil water conditions [8] and soil compaction [39]. The diameter of buckwheat plants was compatible or smaller than these studies, indicating that this method was applicable to these crop plants.

Secondly, the root surface area was calculated accurately from the root specimen (Fig. 4). However, as Kano-Nakata et al. [30, 51] reported, the root length measured from the root specimen was underestimated because of the overlapping roots. This study also underestimated the root length, while the average root diameter was overestimated due to the overlapping of roots (Additional files 4 and 5: Figures S3, S4). Yet, researchers can measure root length and other traits by spreading out the roots on a Plexiglas tray filled with enough tap water at any time, if they stored the root specimen with ethanol or formalin-acetic acid-alcohol solution (FAA). The method developed by Kono et al. [27] requires a folio of the sheets with 960 holes of 5 mm diameter. These holes made the root analysis difficult, as the image analysis detects a hole with a diameter of 5 mm as a root. Therefore, the root specimens were usually dyed with Coomassie Brilliant Blue R to get an optimum contrast [27, 51]. In this study, the holes generated by the root sampling equipment were tiny, and the projected area and the diameter was around 0.014 cm^2^ and 0.67 mm, respectively. These holes can be eliminated using the debris filters by the image preprocessing before analysis. Therefore, the other root staining methods can be applied to analyze the interaction between soil and the physiological function of RSA.

The root specimen sandwiched between the sheets was preserved for more than one year in a refrigerator (6–8 °C) when the whole root specimen was sprayed with 70% ethanol and kept in plastic bags with a zipper. If the researchers need to observe anatomical features of the root, the specimen should be sprayed with FAA instead of ethanol. If soil remains at the base of the root specimen and the root specimen is not preserved in a refrigerator, it decays from the bottom. In another study, buckwheat root specimen with soil on the root base deteriorated in 4 months.

Thirdly, staining of root specimen enabled us to analyze the root-AM fungal interaction (Fig. 3). Though the establishment of AM fungal colonization from spores requires more than nine days under optimal conditions [52, 58], this study assured a reasonably long experimental duration for AM colonization, 52 days. The percentage of root colonization by AM hyphae of the lower-left roots of Nattoukotsubu was 100% (Fig. 3e). In this study, AM fungi colonized locally in the root system, though the soil and AM fungal inoculum were mixed thoroughly. In addition, there was no trend in the manner of AM colonization across replications (data not shown). These results indicated that AM inoculum potential might be low to colonize the whole root system in this study, though positive and negative plant growth responses were observed (Table 1). Since a meta-analysis of studies on plant responses to AM fungi reported the unstable influence of AM fungi on plants [59–66], this type of phenomenon may have occurred in both unpublished and published studies using AM fungi. Yano et al. [48] reported that the root morphological responses to AM inoculation were apparent after 30 days but not after 20 days. This study ensured the cultivation duration and demonstrated the localized root proliferation (Fig. 0.3). The root developed well where AM colonization was high (Additional file 6: Figure S5). It has been reported for several species that lateral root induction in response to AM colonization (reviewed in [14, 67]). Yano et al. [48] reported a localized response of lateral root formation to AM colonization using the revised pinboard method. They clearly showed that AM inoculation of one half of a split-root system of peanut and pigeon pea resulted in a higher number of lateral roots in the inoculated half compared to the non-inoculated half. These root system architectural changes in response to AM colonization were hypothesized to be regulated at least two levels: by their induction before or after the establishment of AM colonization [67], but the molecular mechanisms have not been fully understood. The present method enabled us to sample live root specimens from the root box with minimum damage and displacement of RSA and to observe AM hyphae, which are thinner than the roots (Additional file 7: Figure S6). Though the total sampling time of this method was still long: 7 min, the time required for root washing can be shortened using sand. Combining the improvement of this method with gene expression analysis will reveal the molecular mechanisms of the localized root proliferation.

Phosphorus acquisition efficiency of the root was altered by AM colonization, and it was different between cultivars: it was considerably enhanced in Nattokotsubu, but not much in Tachinagaha (Table 1). There are two known pathways for plant phosphorus uptake from soil: via root epidermal cells (direct pathway) or associations with AM fungi (mycorrhizal pathway), and the two pathways interact in a complex manner [68]. In this study, the roots were only locally colonized by AM fungi. The cultivars responded differently to AM fungi, indicating that each cultivar was affected differently by AM fungi controlling the direct pathway and the mycorrhizal pathway, which eventually affected the shoot response. It should be further studied.

The root specimen of soybean also made it possible to observe the distribution of root nodules (Fig. 3). Kono et al. [8] showed the direct response of root nodulation of three different irrigation methods: the boxes were submerged into the water once a week and allowed to drain to field capacity (control conditions), or the decreased weight of water was added from the surface (surface-irrigated conditions) or the bottom (bottom-irrigated conditions). They showed that the nodules were distributed evenly in the root system under control conditions but concentrated at the upper part of the root system under surface-irrigated conditions and the lower part under bottom-irrigated conditions. Further, Iijima et al. [43, 44] examined the effect of crack fertilization on soybean root nodulation. They found that crack fertilization increased the nodule number in the root system, especially in the lower half, and the increment of nodules accelerated acetylene reduction activity [43] and ureide concentration in xylem sap [44]. The findings were consistently shown under field conditions [43, 44]. The interaction between the other microorganisms such as *Polymyxa* sp. and *Streptomyces mutabilis* and root systems were also reported using the revised pinboard method [42, 45]. Thereby, the present method can be used to analyze plant-microorganism interactions.

This method also provided an opportunity to study the physiological root responses of buckwheat plants to waterlogging stress. Koyama et al. [54] reported that the growth and yield of buckwheat plants were significantly depressed, when the waterlogging treatment duration became longer. Same growth depression was also observed in this study. The shoot dry weight decreased significantly as the flooding period increased (Table 2). Moreover, this study made it possible to sample the living root specimen from the soil with minimum injury at the root tips (Fig. 5). TTC staining of this specimen allowed the measurement of respiratory activity of each root at different depths. The number of TTC stained root tips decreased as the waterlogging treatment duration became longer (Table 2). The xylem sap also showed similar decreasing trends. It has been confirmed that xylem sap ceases, when root systems are subjected to inadequate aeration, low temperature, dry soil, or an inadequate supply of minerals [69]. Further, Yamaguchi et al. [70] reported that root respiration is closely related to xylem sap, and both root respiration and xylem sap become indicators of root activity in rice plants. The whole root specimen of buckwheat plants clearly showed that the root distribution was modified by waterlogging: root development of the lower half was strongly inhibited, as the waterlogging treatment duration became longer (Fig. 5). It may be because buckwheat plants do not develop aerenchyma [71].

In this study, soybean and buckwheat were cultivated for 52 and 31 days in the root boxes, respectively. Kono et al. [27] reported that the root systems of most of the summer cereals and the summer legume fill the root boxes for 30 and 40 days, respectively. Yamauchi et al. [10] cultivated the winter cereals: barley, wheat, rye, and oat for around 130 days from November to March. Thus, this method allows these plant species to grow for a relatively long time. However, it is recommended to determine the growth period in advance by observing the root system development in the box for each crop species.

## Conclusions

The whole root specimen sampled with the revised pinboard method has been used to analyze the interaction between the root system and several soil factors such as soil moisture, soil compaction, microorganism, and allelopathic substances. Also, some of the findings have been consistently expressed under field conditions. This study developed root system sampling equipment and showed that (1) it reduced sampling time almost in half compared to the revised pinboard method and allowed the production of the whole root specimen with minimum injury and displacement of the RSA of soybean and buckwheat, (2) root surface area of soybean plants were calculated accurately from the root specimen, (3) staining of whole root specimen enabled to analyze root-soil interaction in detail: the root response of two soybean cultivars to AM inoculation and the effect of waterlogging stress on the physiological activity of a root system of buckwheat. Using the methods, the evaluation of root traits will contribute to developing agronomic management and introducing root phenotypic traits into the breeding program.

Our method requires no large-scale equipment relative to other two dimensional root phenotyping platforms (Figs. 1, 2). Moreover, the planar optodes have already enabled to map physical properties, such as O_2_, CO_2_, and pH, of soil in root boxes [72, 73]. The accumulation of the multidimensional visual information of whole root specimens and the detailed spatial mapping data of soil environment will further accelerate our understanding of the interaction between RSA and soil environment.

## Supplementary Information

**Additional file 1: Figure S1.** Root system sampling procedure. The letters (a) to (f) correspond to the equipment shown in Fig. 2.

**Additional file 2:Movie S1.** Maize root system sampling procedure.

**Additional file 3**: **Figure S2.** Air temperature and relative humidity of experiment 1 (a) and 2 (b). These weather data were cited from the nearest observatory of each experimental site (Aerological Observatory in Tsukuba, Ibaraki, Japan (a) and Utsunomiya local meteorological office in Utsunomiya, Tochigi, Japan (b), Japan Meteorological Agency, www.data.jma.go.jp).

**Additional file 4**: **Figure S3.** The images analyzed with WinRHIZO (WinRHIZO Pro 2017, Regent Instruments, Inc., Quebec City, QC, Canada): (a,c) the root specimens of soybean plants; (b,d) the roots spread out in water. The roots of a and c were spread out in water as b and d, respectively. The root surface area, root length, and root diameter were shown in Figs. 4 and S4.

**Additional file 5**: **Figure S4.** Correlation between (a) root length calculated from the root specimen of soybean plants and root length calculated from the root spread out on a Plexiglas tray filled with enough tap water, and (b) root diameter calculated from root specimen of soybean plants and root diameter calculated from the root spread out on a Plexiglas tray filled with enough tap water. Both data were obtained from the rectangular sheets cut from whole root specimens as shown in Fig. 3 e and f. The dotted line indicates a one-to-one line. The images of ▲ and ◆ were analyzed with WinRHIZO and shown in Figure S3.

**Additional file 6:Figure S5.** Correlation between root colonization by AM hyphae and the deviation of root surface area in each of the nine rectangular sheets: the results of AM roots of Nattoukotsubu (a) and AM roots of Tachinagaha (b). Deviations are calculated from the following formula: (average of root surface area of 4 replications in each location) – (root surface area of each replication in each location). The data were obtained from the rectangular sheets cut from the whole root specimens as shown in Fig. 3e and f.

**Additional file 7**: **Figure S6.** AM whole root specimens and AM root-external hyphae of two soybean cultivars: Nattoukotsubu (a) and Tachinagaha (b). The frame on the whole root specimens indicates the location of the close-up section. Arrows indicate AM root-external hyphae.

**Additional file 8**: **Figure S7.** Effects of different duration of waterlogging stress on root length distribution in diameter classes of buckwheat plants. C, control plant; W3, 3-day long waterlogged plant; and W6, 6-day long waterlogged plant. One represented plant for each treatment was shown.

**Additional file 9**: **Figure S8.** Correlation between the number of TTC stained root tips and xylem sap of buckwheat plants.

## Data Availability

The datasets used and analyzed during the current study are available from the corresponding author on reasonable request.

## Abbreviations

AM: Arbuscular mycorrhizal
FAA: Formalin-acetic acid-alcohol solution
LR: Lateral root
NM: Non-inoculated
PVC: Polyvinyl chloride
QTL: Quantitative trait locus
RSA: Root system architecture
TTC: 2,3,5-Triphenyl-2*H*-tetrazolium chloride
