# Supplementary figures and images for: Complete root specimen of plants grown in soil-filled root box: sampling, measuring, and staining method
Source: Plant Methods. 2021 Sep 20;17:97. doi: 10.1186/s13007-021-00798-3 (PMC8454053; doi:10.1186/s13007-021-00798-3)

## Slide 1
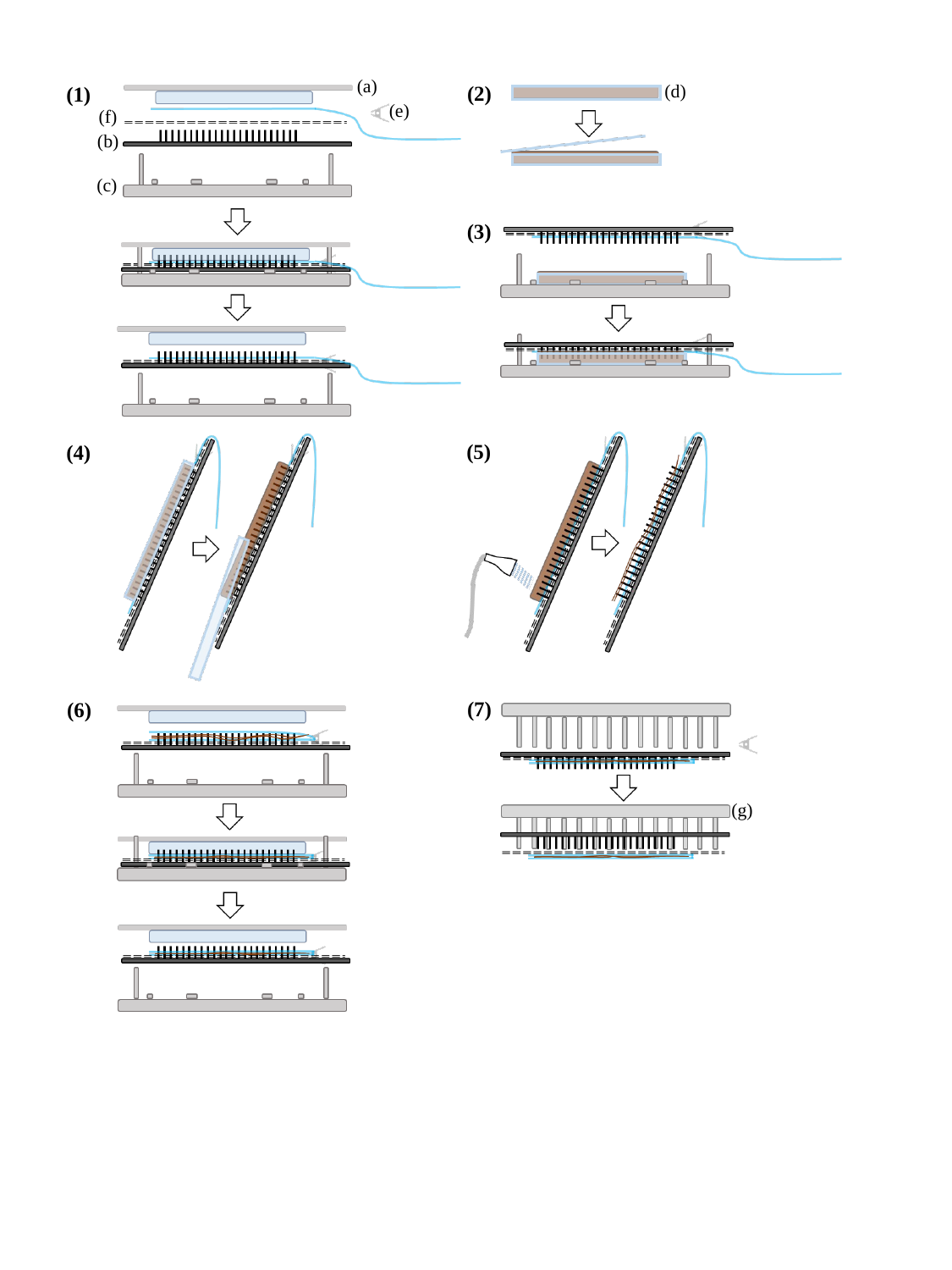

(a)
(d)
(2)
(1)
(e)
(f)
(b)
(c)
(3)
(5)
(4)
(7)
(6)
(g)

Supplement: Supplementary file 1 — Additional file 1: Figure S1. Root system sampling procedure. The letters (a) to (f) correspond to the equipment shown in Fig. 2. [file 13007_2021_798_MOESM1_ESM.pptx]

## Slide 1
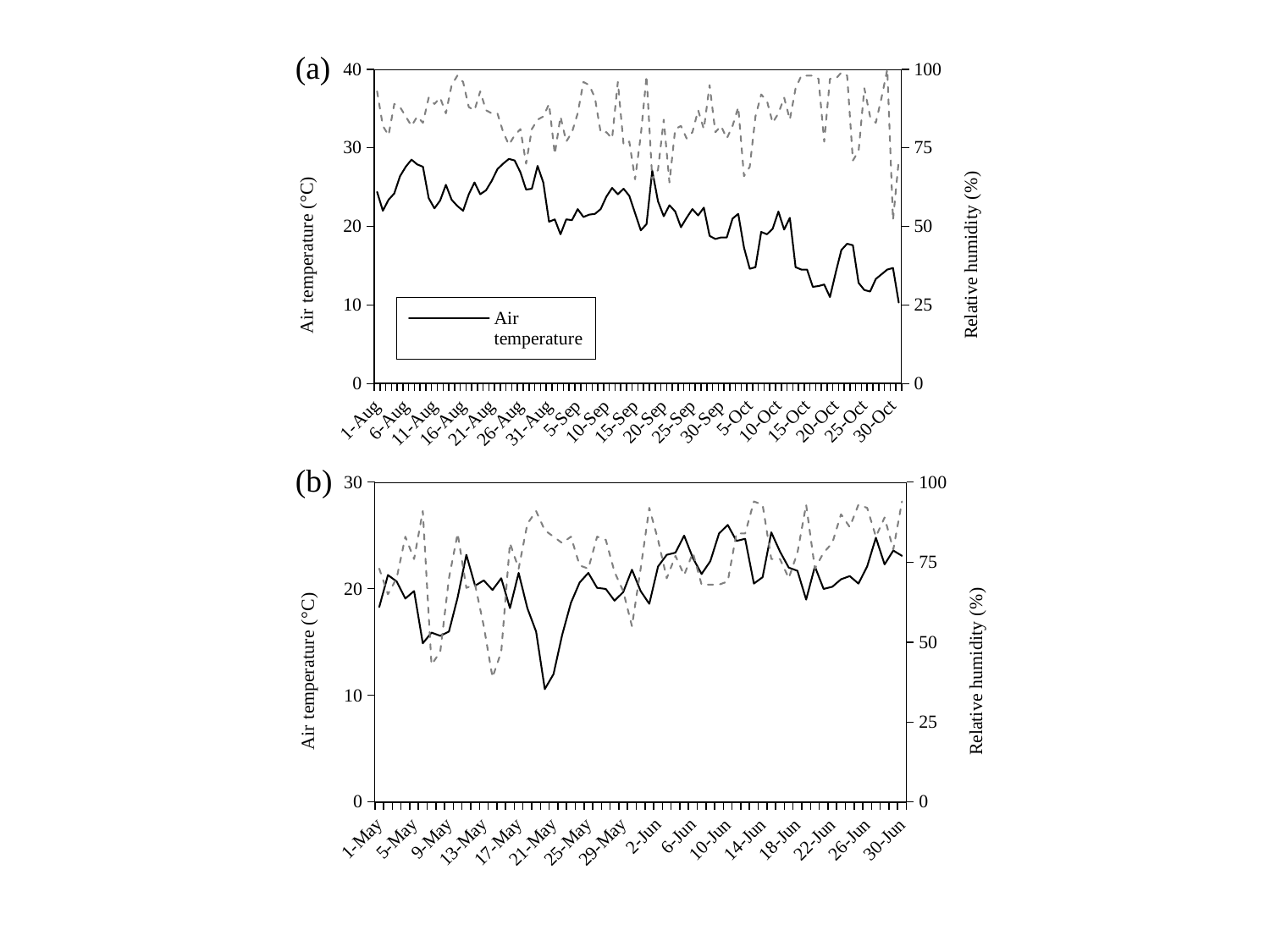

(a)
### Chart
| Category | | |
|---|---|---|
| 42948 | 24.4 | 93.0 |
| 42949 | 22.0 | 82.0 |
| 42950 | 23.4 | 79.0 |
| 42951 | 24.2 | 89.0 |
| 42952 | 26.4 | 88.0 |
| 42953 | 27.6 | 85.0 |
| 42954 | 28.5 | 82.0 |
| 42955 | 27.9 | 85.0 |
| 42956 | 27.6 | 83.0 |
| 42957 | 23.6 | 91.0 |
| 42958 | 22.3 | 89.0 |
| 42959 | 23.3 | 91.0 |
| 42960 | 25.3 | 86.0 |
| 42961 | 23.4 | 95.0 |
| 42962 | 22.6 | 98.0 |
| 42963 | 22.0 | 96.0 |
| 42964 | 24.1 | 88.0 |
| 42965 | 25.6 | 87.0 |
| 42966 | 24.1 | 93.0 |
| 42967 | 24.6 | 87.0 |
| 42968 | 25.8 | 86.0 |
| 42969 | 27.3 | 86.0 |
| 42970 | 28.0 | 80.0 |
| 42971 | 28.6 | 76.0 |
| 42972 | 28.4 | 79.0 |
| 42973 | 26.9 | 81.0 |
| 42974 | 24.7 | 70.0 |
| 42975 | 24.8 | 81.0 |
| 42976 | 27.7 | 84.0 |
| 42977 | 25.6 | 85.0 |
| 42978 | 20.6 | 89.0 |
| 42979 | 20.9 | 73.0 |
| 42980 | 19.0 | 85.0 |
| 42981 | 20.9 | 77.0 |
| 42982 | 20.8 | 80.0 |
| 42983 | 22.2 | 86.0 |
| 42984 | 21.2 | 96.0 |
| 42985 | 21.5 | 95.0 |
| 42986 | 21.6 | 91.0 |
| 42987 | 22.2 | 80.0 |
| 42988 | 23.8 | 80.0 |
| 42989 | 24.9 | 78.0 |
| 42990 | 24.1 | 96.0 |
| 42991 | 24.8 | 76.0 |
| 42992 | 23.9 | 77.0 |
| 42993 | 21.7 | 65.0 |
| 42994 | 19.5 | 79.0 |
| 42995 | 20.3 | 98.0 |
| 42996 | 27.1 | 65.0 |
| 42997 | 23.2 | 68.0 |
| 42998 | 21.3 | 84.0 |
| 42999 | 22.7 | 64.0 |
| 43000 | 21.9 | 81.0 |
| 43001 | 19.9 | 82.0 |
| 43002 | 21.1 | 78.0 |
| 43003 | 22.2 | 80.0 |
| 43004 | 21.4 | 87.0 |
| 43005 | 22.4 | 81.0 |
| 43006 | 18.8 | 95.0 |
| 43007 | 18.4 | 80.0 |
| 43008 | 18.6 | 82.0 |
| 43009 | 18.6 | 78.0 |
| 43010 | 21.0 | 82.0 |
| 43011 | 21.6 | 88.0 |
| 43012 | 17.3 | 66.0 |
| 43013 | 14.6 | 69.0 |
| 43014 | 14.8 | 85.0 |
| 43015 | 19.3 | 92.0 |
| 43016 | 19.0 | 90.0 |
| 43017 | 19.7 | 83.0 |
| 43018 | 21.9 | 86.0 |
| 43019 | 19.6 | 91.0 |
| 43020 | 21.1 | 84.0 |
| 43021 | 14.8 | 94.0 |
| 43022 | 14.5 | 98.0 |
| 43023 | 14.5 | 98.0 |
| 43024 | 12.3 | 98.0 |
| 43025 | 12.4 | 97.0 |
| 43026 | 12.6 | 77.0 |
| 43027 | 11.0 | 97.0 |
| 43028 | 14.1 | 97.0 |
| 43029 | 17.0 | 99.0 |
| 43030 | 17.8 | 98.0 |
| 43031 | 17.6 | 71.0 |
| 43032 | 12.8 | 74.0 |
| 43033 | 11.9 | 94.0 |
| 43034 | 11.7 | 85.0 |
| 43035 | 13.3 | 83.0 |
| 43036 | 13.9 | 91.0 |
| 43037 | 14.5 | 100.0 |
| 43038 | 14.7 | 52.0 |
| 43039 | 10.3 | 71.0 |(b)
### Chart
| Category | | |
|---|---|---|
| 43952 | 18.3 | 73.0 |
| 43953 | 21.3 | 65.0 |
| 43954 | 20.7 | 70.0 |
| 43955 | 19.1 | 83.0 |
| 43956 | 19.8 | 76.0 |
| 43957 | 14.9 | 91.0 |
| 43958 | 15.9 | 43.0 |
| 43959 | 15.6 | 47.0 |
| 43960 | 16.0 | 70.0 |
| 43961 | 19.2 | 84.0 |
| 43962 | 23.2 | 67.0 |
| 43963 | 20.3 | 68.0 |
| 43964 | 20.8 | 55.0 |
| 43965 | 19.9 | 39.0 |
| 43966 | 21.0 | 47.0 |
| 43967 | 18.2 | 81.0 |
| 43968 | 21.5 | 73.0 |
| 43969 | 18.2 | 87.0 |
| 43970 | 16.0 | 91.0 |
| 43971 | 10.6 | 85.0 |
| 43972 | 12.0 | 83.0 |
| 43973 | 15.7 | 81.0 |
| 43974 | 18.7 | 83.0 |
| 43975 | 20.6 | 74.0 |
| 43976 | 21.5 | 73.0 |
| 43977 | 20.1 | 83.0 |
| 43978 | 20.0 | 82.0 |
| 43979 | 18.9 | 72.0 |
| 43980 | 19.7 | 66.0 |
| 43981 | 21.8 | 55.0 |
| 43982 | 19.8 | 73.0 |
| 43983 | 18.6 | 92.0 |
| 43984 | 22.1 | 82.0 |
| 43985 | 23.2 | 70.0 |
| 43986 | 23.4 | 77.0 |
| 43987 | 25.0 | 71.0 |
| 43988 | 22.9 | 78.0 |
| 43989 | 21.4 | 68.0 |
| 43990 | 22.6 | 68.0 |
| 43991 | 25.2 | 68.0 |
| 43992 | 26.0 | 69.0 |
| 43993 | 24.5 | 84.0 |
| 43994 | 24.7 | 84.0 |
| 43995 | 20.5 | 94.0 |
| 43996 | 21.1 | 93.0 |
| 43997 | 25.3 | 76.0 |
| 43998 | 23.5 | 76.0 |
| 43999 | 22.0 | 70.0 |
| 44000 | 21.7 | 78.0 |
| 44001 | 19.0 | 93.0 |
| 44002 | 22.1 | 73.0 |
| 44003 | 20.0 | 78.0 |
| 44004 | 20.2 | 81.0 |
| 44005 | 20.9 | 90.0 |
| 44006 | 21.2 | 86.0 |
| 44007 | 20.5 | 93.0 |
| 44008 | 22.1 | 92.0 |
| 44009 | 24.8 | 83.0 |
| 44010 | 22.3 | 89.0 |
| 44011 | 23.6 | 79.0 |
| 44012 | 23.1 | 94.0 |

Supplement: Supplementary file 3 — Additional file 3: Figure S2. Air temperature and relative humidity of experiment 1 (a) and 2 (b). These weather data were cited from the nearest observatory of each experimental site (Aerological Observatory in Tsukuba, Ibaraki, Japan (a) and Utsunomiya local meteorological office in Utsunomiya, Tochigi, Japan (b), Japan Meteorological Agency, www.data.jma.go.jp). [file 13007_2021_798_MOESM3_ESM.pptx]

## Slide 1
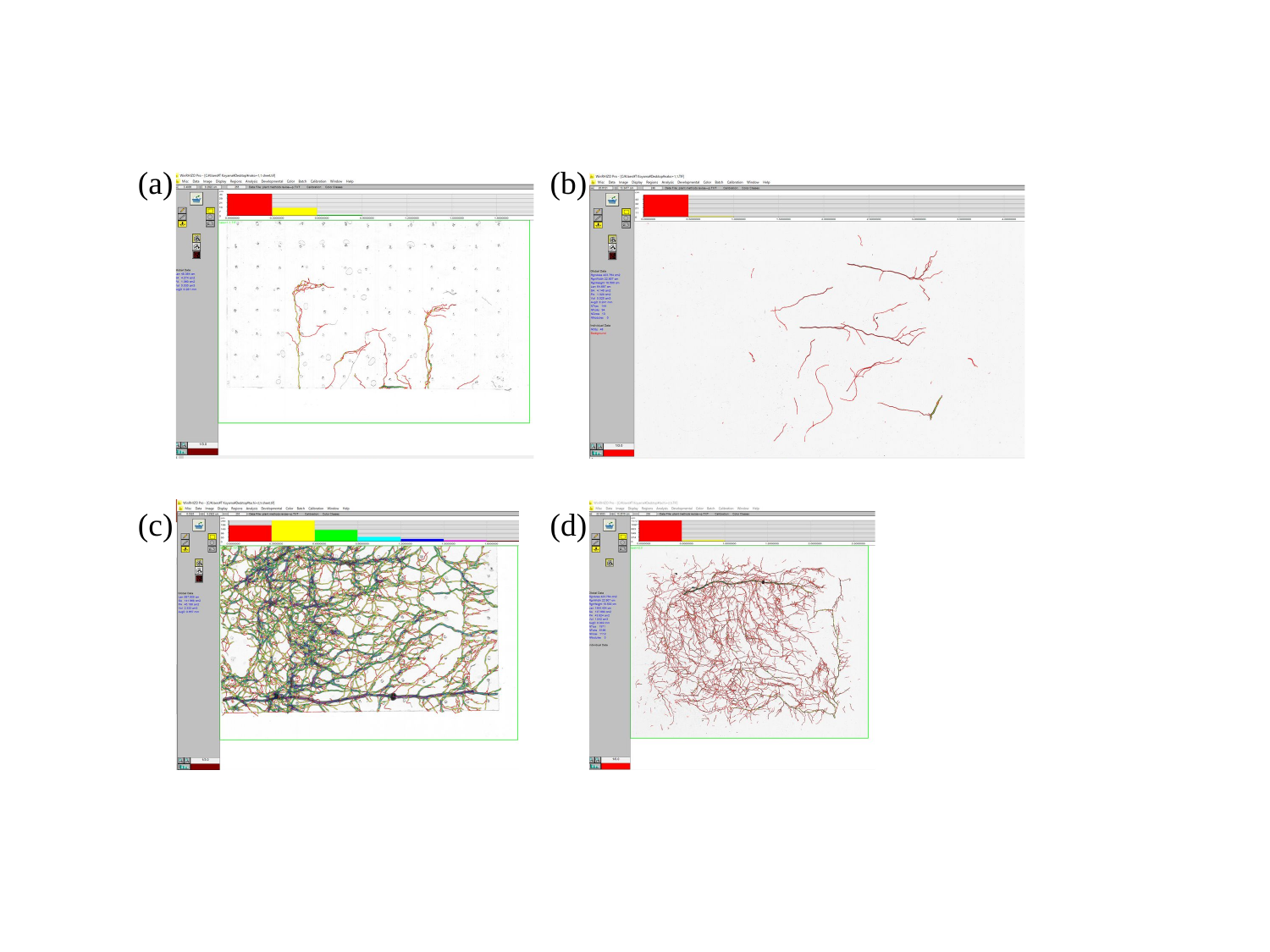

(a)
(b)
(c)
(d)

Supplement: Supplementary file 4 — Additional file 4: Figure S3. The images analyzed with WinRHIZO (WinRHIZO Pro 2017, Regent Instruments, Inc., Quebec City, QC, Canada): (a,c) the root specimens of soybean plants; (b,d) the roots spread out in water. The roots of a and c were spread out in water as b and d, respectively. The root surface area, root length, and root diameter were shown in Figs. 4 and S4. [file 13007_2021_798_MOESM4_ESM.pptx]

## Slide 1
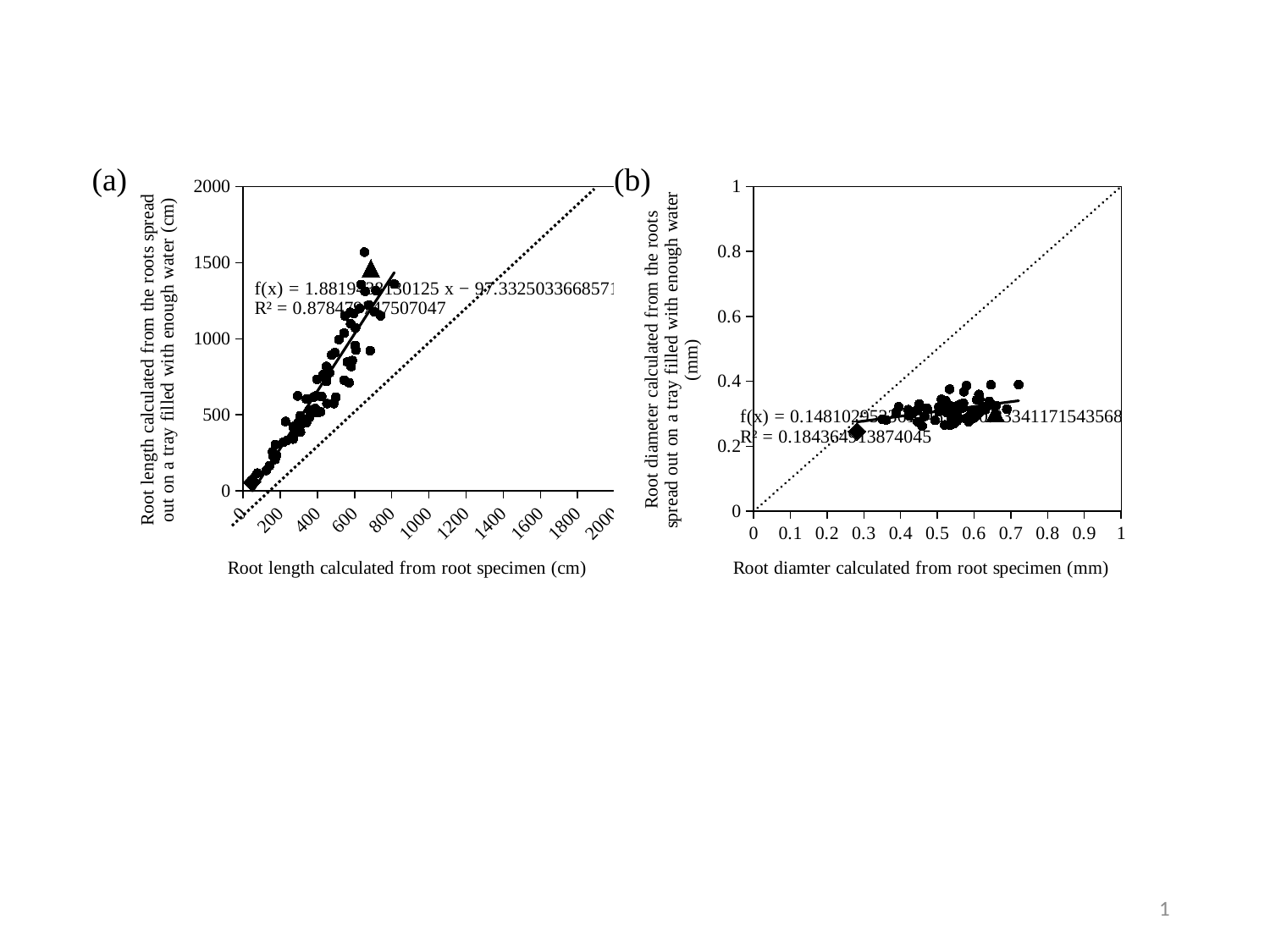

(a)
(b)
### Chart
| Category | | |
|---|---|---|
### Chart
| Category | | |
|---|---|---|1

Supplement: Supplementary file 5 — Additional file 5: Figure S4. Correlation between (a) root length calculated from the root specimen of soybean plants and root length calculated from the root spread out on a Plexiglas tray filled with enough tap water, and (b) root diameter calculated from root specimen of soybean plants and root diameter calculated from the root spread out on a Plexiglas tray filled with enough tap water. Both data were obtained from the rectangular sheets cut from whole root specimens as shown in Fig. 3 e and f. The dotted line indicates a one-to-one line. The images of ▲ and ◆ were analyzed with WinRHIZO and shown in Figure S3. [file 13007_2021_798_MOESM5_ESM.pptx]

(a)

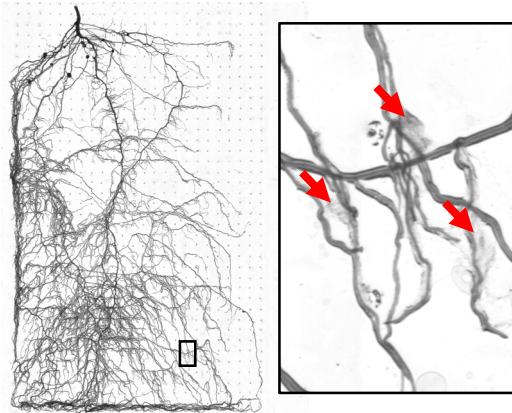

(b)

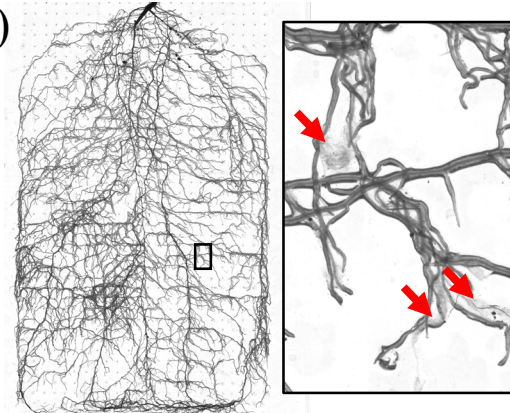

Supplement: Supplementary file 7 — Additional file 7: Figure S6. AM whole root specimens and AM root-external hyphae of two soybean cultivars: Nattoukotsubu (a) and Tachinagaha (b). The frame on the whole root specimens indicates the location of the close-up section. Arrows indicate AM root-external hyphae. [file 13007_2021_798_MOESM7_ESM.pdf]

## Slide 1
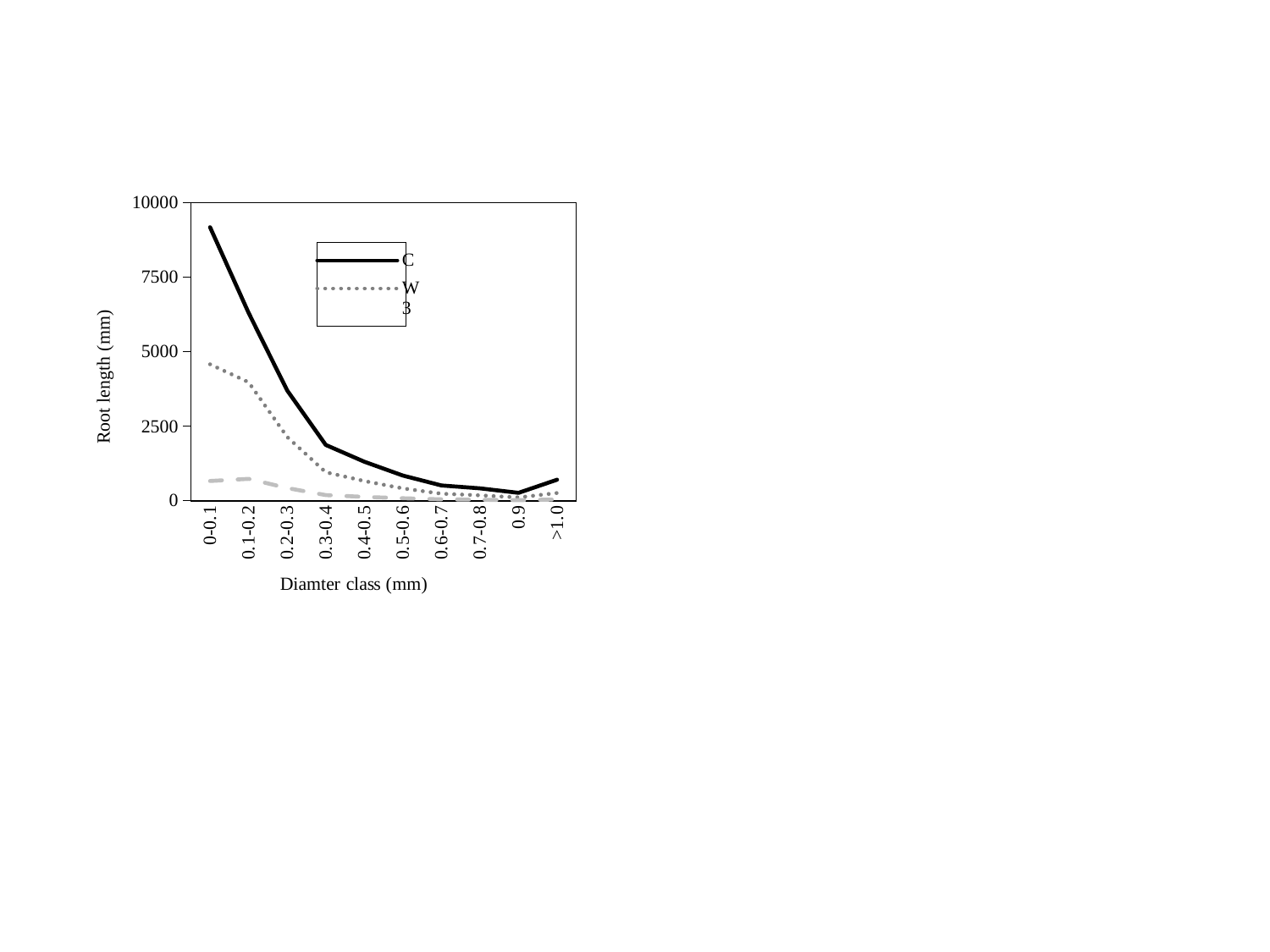

### Chart
| Category | C | W3 | W6 |
|---|---|---|---|
| 0-0.1 | 9182.2764 | 4582.417900000001 | 665.2122 |
| 0.1-0.2 | 6308.548499999999 | 3979.2414999999996 | 736.1841 |
| 0.2-0.3 | 3705.5661 | 2142.3467 | 428.27340000000004 |
| 0.3-0.4 | 1874.6679000000001 | 958.4477999999999 | 191.87329999999997 |
| 0.4-0.5 | 1311.8561 | 662.3709 | 131.9806 |
| 0.5-0.6 | 847.7950999999999 | 416.2833000000001 | 86.50999999999999 |
| 0.6-0.7 | 515.1095 | 238.32730000000004 | 49.1129 |
| 0.7-0.8 | 418.951 | 183.0271 | 35.9409 |
| 0.9 | 267.85940000000005 | 113.9492 | 23.467999999999996 |
| >1.0 | 707.9789999999999 | 261.99370000000005 | 43.6931 |

Supplement: Supplementary file 8 — Additional file 8: Figure S7. Effects of different duration of waterlogging stress on root length distribution in diameter classes of buckwheat plants. C, control plant; W3, 3-day long waterlogged plant; and W6, 6-day long waterlogged plant. One represented plant for each treatment was shown. [file 13007_2021_798_MOESM8_ESM.pptx]

## Slide 1
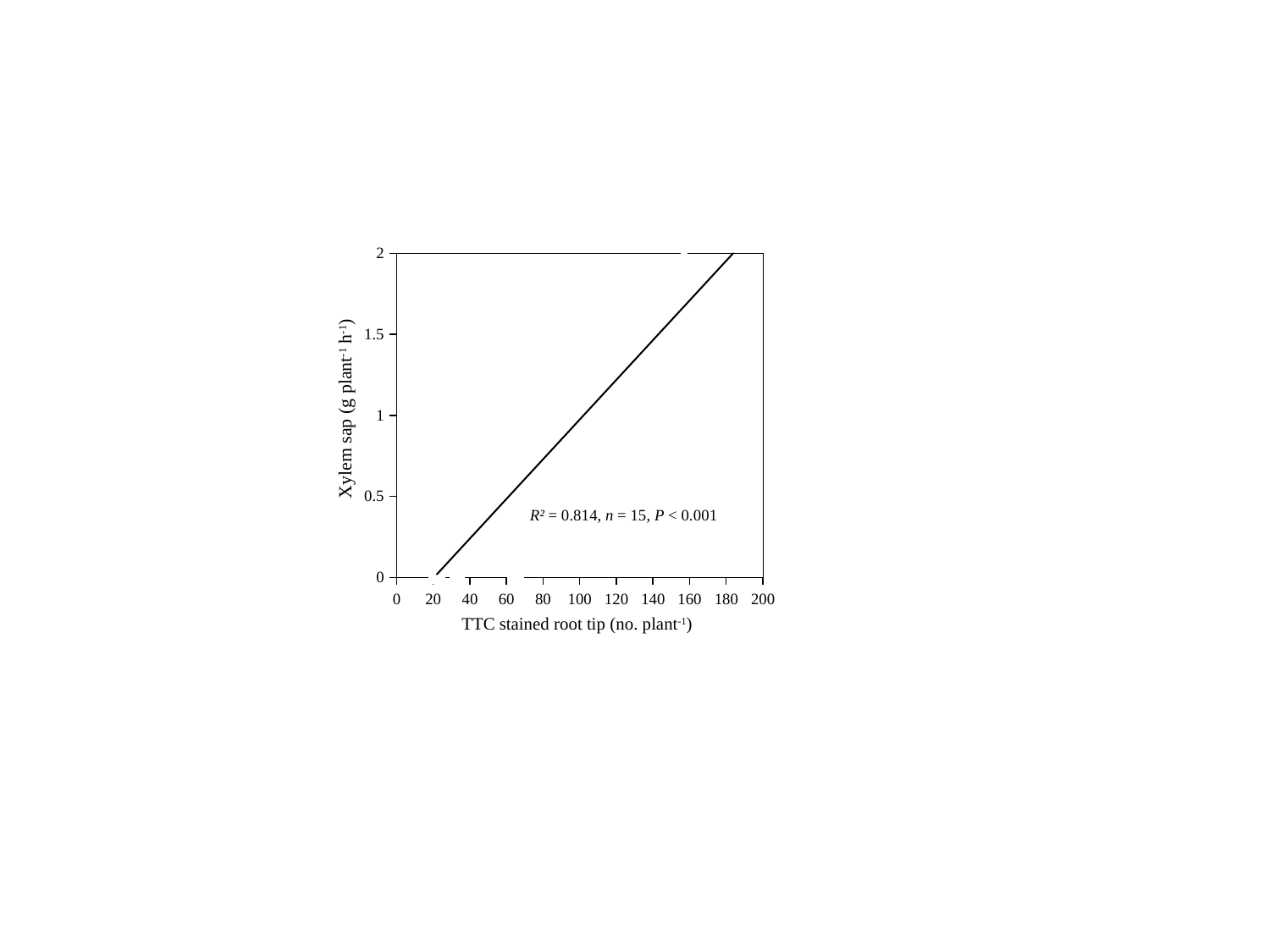

### Chart
| Category | | | | |
|---|---|---|---|---|Xylem sap (g plant-1 h-1)
R² = 0.814, n = 15, P < 0.001
TTC stained root tip (no. plant-1)

Supplement: Supplementary file 9 — Additional file 9: Figure S8. Correlation between the number of TTC stained root tips and xylem sap of buckwheat plants. [file 13007_2021_798_MOESM9_ESM.pptx]
