# Supplementary material for: Complete root specimen of plants grown in soil-filled root box: sampling, measuring, and staining method
Source: Plant Methods. 2021 Sep 20;17:97. doi: 10.1186/s13007-021-00798-3 (PMC8454053; doi:10.1186/s13007-021-00798-3)
Supplement: Supplementary file 6 — Additional file 6:Figure S5. Correlation between root colonization by AM hyphae and the deviation of root surface area in each of the nine rectangular sheets: the results of AM roots of Nattoukotsubu (a) and AM roots of Tachinagaha (b). Deviations are calculated from the following formula: (average of root surface area of 4 replications in each location) – (root surface area of each replication in each location). The data were obtained from the rectangular sheets cut from the whole root specimens as shown in Fig. 3e and f. [file 13007_2021_798_MOESM6_ESM.pptx]

## Slide 1
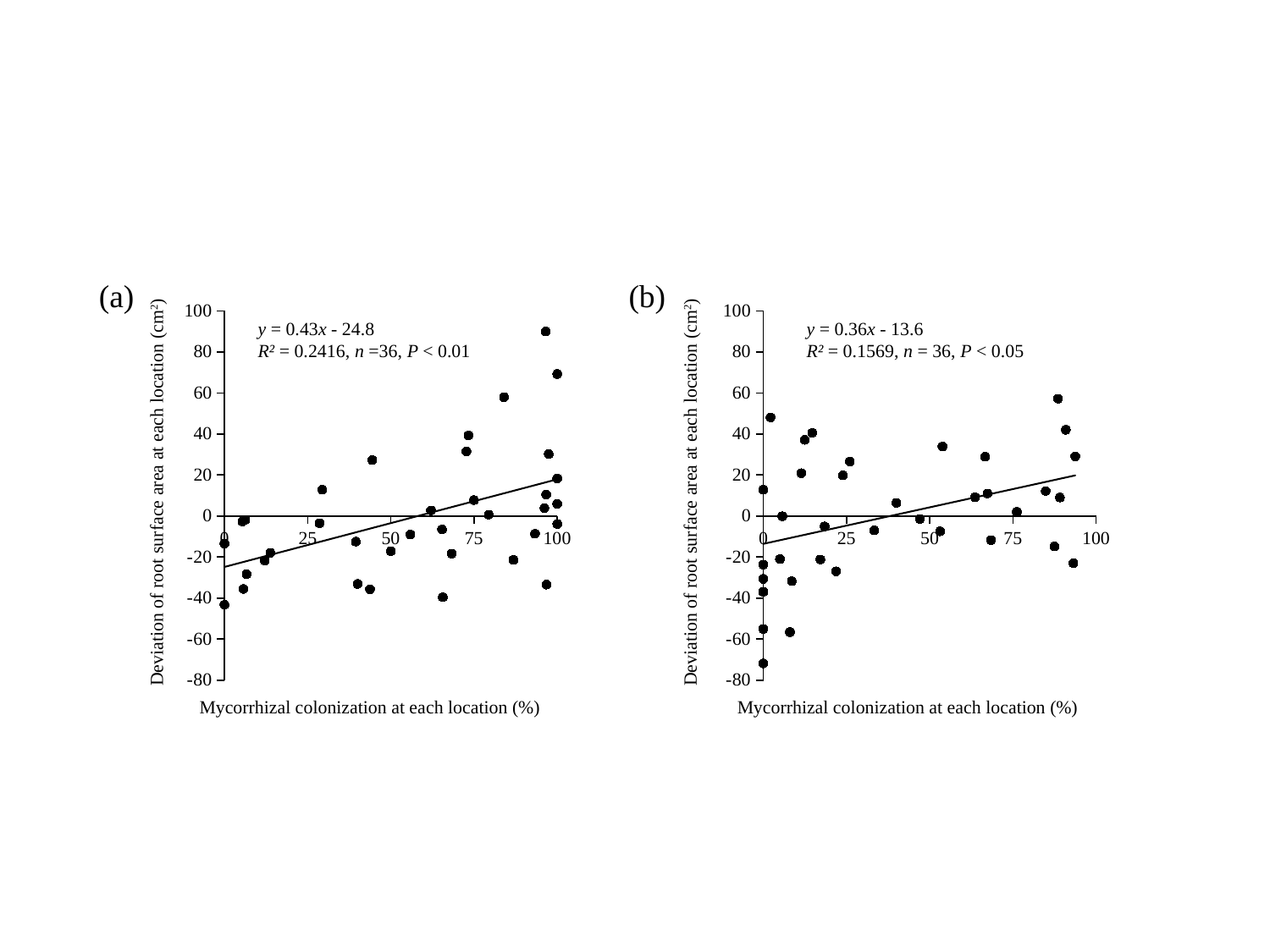

(a)
(b)
### Chart
| Category | |
|---|---|
### Chart
| Category | |
|---|---|y = 0.43x - 24.8
R² = 0.2416, n =36, P < 0.01
y = 0.36x - 13.6
R² = 0.1569, n = 36, P < 0.05
Deviation of root surface area at each location (cm2)
Deviation of root surface area at each location (cm2)
Mycorrhizal colonization at each location (%)
Mycorrhizal colonization at each location (%)
